# Chemical datuments as scientific enablers

**DOI:** 10.1186/1758-2946-5-6

**Published:** 2013-01-23

**Authors:** Henry S Rzepa

**Affiliations:** 1 Department of Chemistry, Imperial College London, South Kensington Campus, London, UK

## Abstract

This article is an attempt to construct a chemical datument as a means of presenting insights into chemical phenomena in a scientific journal. An exploration of the interactions present in a small fragment of duplex Z-DNA and the nature of the catalytic centre of a carbon-dioxide/alkene epoxide alternating co-polymerisation is presented in this datument, with examples of the use of three software tools, one based on Java, the other two using Javascript and HTML5 technologies. The implications for the evolution of scientific journals are discussed.

## Background

Chemical sciences are often considered to stand at the crossroads of paths to many disciplines, including molecular and life sciences, materials and polymer sciences, physics, mathematical and computer sciences. As a research discipline, chemistry has itself evolved over the last few decades to focus its metaphorical microscope on both far larger and more complex molecular systems than previously attempted, as well as uncovering a far more subtle understanding of the quantum mechanical underpinnings of even the smallest of molecules. Both these extremes, and everything in between, rely heavily on data. Data in turn is often presented in the form of visual or temporal models that are constructed to illustrate molecular behaviour and the scientific semantics. In the present article, I argue that the mechanisms for sharing both the underlying data, and the (semantic) models between scientists need to evolve in parallel with the increasing complexity of these models. Put simply, the main exchange mechanism, the scientific journal, is accepted [1] as seriously lagging behind in its fitness for purpose. It is in urgent need of reinvention; one experiment in such was presented as a data-rich chemical exploratorium [2]. My case here in this article will be based on my recent research experiences in two specific areas. The first involves a detailed analysis of the inner kernel of the Z-DNA duplex using modern techniques for interpreting the electronic properties of a molecule. The second recounts the experiences learnt from modelling the catalysed alternating co-polymerisation of an alkene epoxide and carbon dioxide.

An attempt will here be made to present both stories in the form of a chemical datument. This portmanteau word refers to a data-rich document, and is used here to mean a document that describes a story of chemical research in a manner which allows the data underpinning the discourse to be provided as an integral part of that story. Although the term *datument* was originally explicitly coined in a scientific context in 2004 [3], arguably the first true datument on the topic of molecular science was published in a mainstream peer-reviewed chemistry journal had appeared as early as 2001 [4]. This latter article has several unusual attributes. It attracted an editorial comment [5] that describes the article as an "*interesting experiment*", but which also concludes that "*it wasn't easy to deal with by any means*", referring to the production process. In this sense, this article was also arguably ahead of its time, since it required an early beta version of a Web browser to expose the available data to the reader (Internet Explorer 6.0 or 6.5) using a combination of XML as the carrier of the data/content and XSLT stylesheets to transform this for browser presentation. Modern browsers support newer versions of the standards used for these operations and some 11 years on, the original article now needs "maintenance" to recover these aspects. But nevertheless, the data contained with it, expressed in XML and CML [6] as the principle carrier of chemical information retains all of its original semantic meanings, and it is specifically the presentational layer that requires the maintenance. This of itself raises some interesting issues which will need to be addressed in the future. In turn, it may also mean that the presentational mechanisms used in the current article may equally need curation in the future. In the last 11 years nevertheless, made major advances in this area of semantic scientific publishing have been made, and the reader is referred to several excellent reviews of this area for further information [7,8].

### Case 1. The inner secrets of the structure of Z-DNA

In a previous article on the topic [9], I recounted how early papers describing the molecular structure of the DNA double-helix were quite data-impoverished. The issue related to why this molecule adopted a left or right handed helical wind and what the factors influencing this balance might have been. To analyse these features requires evaluating the wavefunction of a (small fragment) of the system. This is then inspected for not only the electronic interactions between covalent bonds themselves but also the nature of any close (non-covalent) contacts between pairs of atoms which do not classify as bonds or appear in a bond connection table (and are therefore un-indexed and hence neglected) [2].

Two analytical tools were utilised and the results are presented here.

1. The first was a so-called Natural-bond-orbital analysis [10,11], the basis of which is to transform the computed wavefunction of the molecule into localised functions called NBOs, which take two basic forms. The first has a two-electron occupancy, and is deemed to be a potential *donor* of these two electrons (BD). The second is an NBO with zero-electron occupancy, and which is deemed to be an acceptor of electrons (BD*). The extent to which the latter influences the former is quantified by a perturbation energy E(2). The magnitude of this term in turn depends on both the difference in energy between the two interacting NBOs and the degree of overlap between them. Whilst the former can be expressed simply by an energy, the latter lends itself to visual presentation as a set of overlapping iso-surfaces.

2. The NBO procedure, by definition, explores how bonds BD interact with anti-bonds, BD*, within a molecule. But almost as important are the regions which conventionally are not defined as bonds, but are instead referred to as non-covalent interactions within a molecule. A hydrogen bond is one example of this type, but they can also refer to weaker interactions. It is important to appreciate that although any single such interaction may be quite weak, repeated occurrence in a large molecule will tend to accumulate the effect. This NCI procedure [12-14] involves computing the reduced (electron) density gradient isosurfaces for the molecule in question, and filtering the range of this value to that which focuses only on the weakly interacting regions. A further property (the density Laplacian) can be used in conjunction with colour coding of the isosurface to indicate whether the interaction is attractive or repulsive. Again, this is a complex visual surface generated from a computed molecular wavefunction.

You might appreciate that communicating these concepts to the reader using merely descriptive text and static diagrams (even the use of colour by authors in diagrams can incur very substantial/additional costs charged by the publisher) may be very limiting. Of course, I have also selected this example for precisely such difficulty, and to introduce how a datument might go a long way towards addressing this problem.

### Case 2. Unravelling the mechanism of co-polymerisations

**Figure 1:**
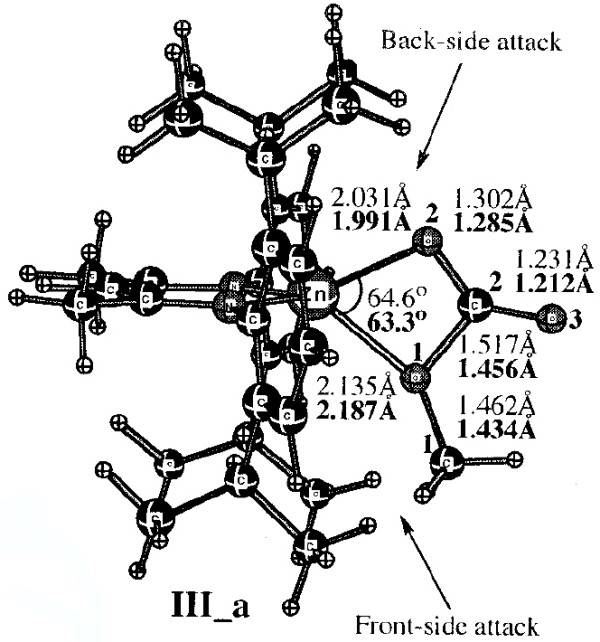
Model for metal catalysed co-polymerisation of epoxide and carbon dioxide showing an intermediate in the mechanism.

**Figure 2:**
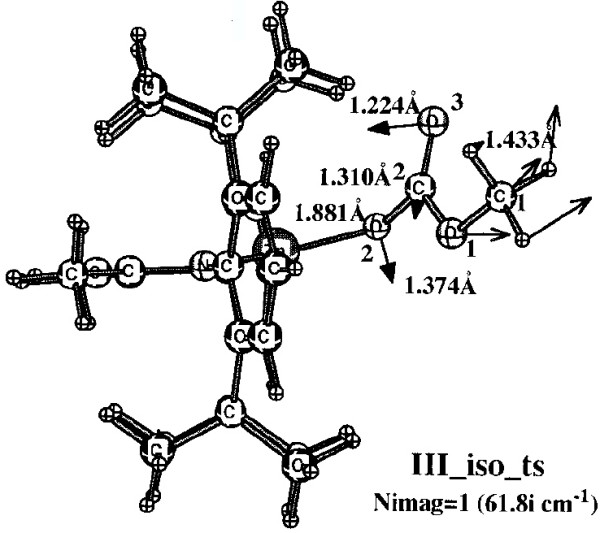
Model for metal catalysed co-polymerisation of epoxide and carbon dioxide showing a transition state in the mechanism.

Optimising the effective use of carbon dioxide as a C_1_ feedstock for manufacturing polymers is a pressing scientific challenge [15]. The answer lies in understanding the complex catalytic chemistries of quite large molecular systems. These chemistries can increasingly be successfully modelled using modern quantum chemical theories. The capability to address these complex catalytic systems first emerged around 2002 when, in something of a tour-de-force for that period, Morokuma and co-workers reported their exploration of the mechanism of Zinc(II)-catalysed alternating co-polymerisation of carbon dioxide with cyclohexene epoxide [16]. The objective then was to develop a rational explanation for why the polymer alternated, *i.e.* by addition of one molecule of carbon dioxide monomer was invariably followed by one molecule of cyclohexene epoxide, and then again by CO_2_. By 2012 the complexity and subtle nature of the challenge had increased, the challenge now being an understanding [17] of how asymmetric induction in the resulting polymer can be achieved. Answering such questions involves a detailed and intricate knowledge of the reacting (covalent or ionic) bonds themselves and (as with the DNA project discussed above) the nature of the non-covalent interactions [12-14]. The model one builds to explore such aspects may contain up to 200 atoms (in a polymer of course, it is potentially much larger, and one has to truncate the model to mangeable proportions). Two examples of this complexity are shown below (Figures 1 and 2). 

Firstly, I should state that this article is typical of the period (a mere ten years ago), whereby limitations on the page length precluded inclusion of any tables of coordinates (=data) associated with this figure. Seeking to explore the attributes of the bonds and appropriate non-covalent interactions requires data. It was however reasonably common in that period, but certainly not mandatory, for such data to be included in the *supporting information* (note it is styled as information, not data). In this particular instance, no such information is actually available. Even in 2011 when another article on this topic was published [18], and for which supporting information was available, one finds a paucity of the type of data required to reconstitute any of the models on which all the assertions in the body of the article are based. Unlike the area of crystallography, where data deposition is mandatory [19], no such requirements exist for many other areas of data-rich chemistry, including *e.g.* for computational chemistry.

The figures themselves (Figure 1) contain information that only a human could attribute meaning to (the figures could not for example be automatically mined for information in the manner that eg the OSCAR project [20] has demonstrated). Even this author (as a human), struggled to reconstitute a usable model from these figures. There is indeed some numerical information associated (note that text mining software cannot access this information) with the figure, styled in *lightface* and *boldface* (each being the result of using a different theoretical method, as explained in the original figure caption). but for a system with perhaps 100 atoms (counting the actual number from the figure is essentially impossible), this represents only 7 of the 294 (=3N-6) variables required to precisely define the three dimensional model. Other annotations are present in the figure; the designation *back-side* or *front-side* attack have a semantic meaning that is not immediately obvious to someone not very familiar with the original research. One of the diagrams (Figure 2) has lines carrying arrow-heads, the other (Figure 1) does not. Although not explained in the figure caption, an experienced (human) computational chemist can nevertheless infer the semantics that these are normal vibrational mode displacement vectors. Furthermore it is likely that this particular mode is selected because it represents the vibration for (harmonic) motion of the atoms at the transition state for the reaction. These vectors may or may not be mass-weighted, so their length may carry no significance. One might associate this feature with the (non-minable) text below it indicating that the (imaginary) wave number of this mode is 61.8i cm^-1^. One might infer that the diagram in which such vectors are absent (Figure 1) is not a transition state, but an equilibrium structure in which all the normal modes are real and not imaginary. That is a lot of implicit semantics, which a (trained) human can cope with, but which again a machine is unlikely to. I have gone into this one figure in some detail, not at all to criticise the authors for providing a deficient figure, but to illustrate the extent to which the data from which the model can be built is lacking, together with the semantics needed to put that data to good use.

The preceding analysis of these articles [16,17,20] originated as an outcome of the exploration of the mechanism of this reaction [15]. In order to compare new models with the earlier ones, it was essential to analyse the information carried in Figures 1 and 2, in particular whether the system shown in these figures represented a mono or a bimetallic system. Because of the particular projection onto two dimensions used in the published figures, it was not possible to establish with absolute certainty whether a second Zn atom might be present, but be obscured in the figure. After about an hour of such analysis, a key (semantic) connection was made, the realisation that bimetallic models had only been explicitly discussed in the literature from the year 2003 onwards. From this chronology, it was possible to conclude that it was probable that the 2002 model did not represent a bimetallic system. Unfortunately, there were no data presented in the form of atom lists which would have instantly clarified this aspect.

## Discussion

When it came to communicating our own researches on these topics [15], it was imperative that we should explore how to not propagate the difficulties we ourselves had experienced with the earlier literature onto future readers of our own article. How could the appropriate data (and semantics) be incorporated into a journal article in 2012? One publisher is already making a virtue of this aspect in advertising *the-article-of-the-future*[21]. These articles feature data-based components such as compound information, experimental flowcharts and embedded video (although potentially data rich, this data can often be inaccessible in the same sense that was discussed for Figures 1 and 2. An animation for example can only be viewed from the author's predetermined viewpoint, and not from the reader's). At the time of writing, there are no examples of articles-of-the-future [21] suitable for describing the type of research discussed above. In fact, we had started an exploration in 2006 [22] of data-rich articles which contained so-called web-enhanced objects (tables and figures) in conjunction with other publishers [23]. These constitute datuments in the sense that not only can a human easily re-use the data carried in such an article, but in theory so could a (much more pedantic) software agent tasked to mine the data. Such mining is facilitated by using XHTML to express the datument (PDF versions of the articles were also made available by the publisher, but the semantic data-enrichment is not present in these versions). These articles however were not optimised for their semantic attributes. Our 2001 datument [4] was expressed entirely in XML, and presented to the reader by an on-the-fly transformation of that XML using appropriate XSLT stylesheets. Our 2006+ web-enhanced figures and tables accepted the practical reality that publishers were not yet ready to accept XML/XSLT submissions, as well as the observation that very few authors had the time and skills to author datuments in this format.

### The digital data repository and data semantics

It is important to distinguish between data and the wrapper by which it is presented to the (human) reader. There are several considerations.

1. The raw unprocessed data may be too large to reasonably include in a datument.

2. Or it may take a lot of processing power, or require complex computer code, to transform the data into a meaningful visual appearance.

3. The raw data may have no meta-data associated with it, and hence may not be semantically processable or searchable.

The expedient adopted here is to include at least sufficient well-structured (i.e. XML-based) data to allow regeneration of the original (large) dataset with almost no effort required to achieve this. If the transformation of the data for visual presentation is itself too complex to be handled by a browser in real-time, then the result of that transform can itself be included in the datument (again ideally as an XML dataset). Finally, to complete the utility of the datument, the (possibly large) inputs and outputs from which the dataset derives can be linked to a digital repository where the semantic enrichment can be added in a largely automatic manner. This in turn would allow either humans or software agents to process them if desired.

#### Examples of digital repositories containing molecular data

**Figure 3:**
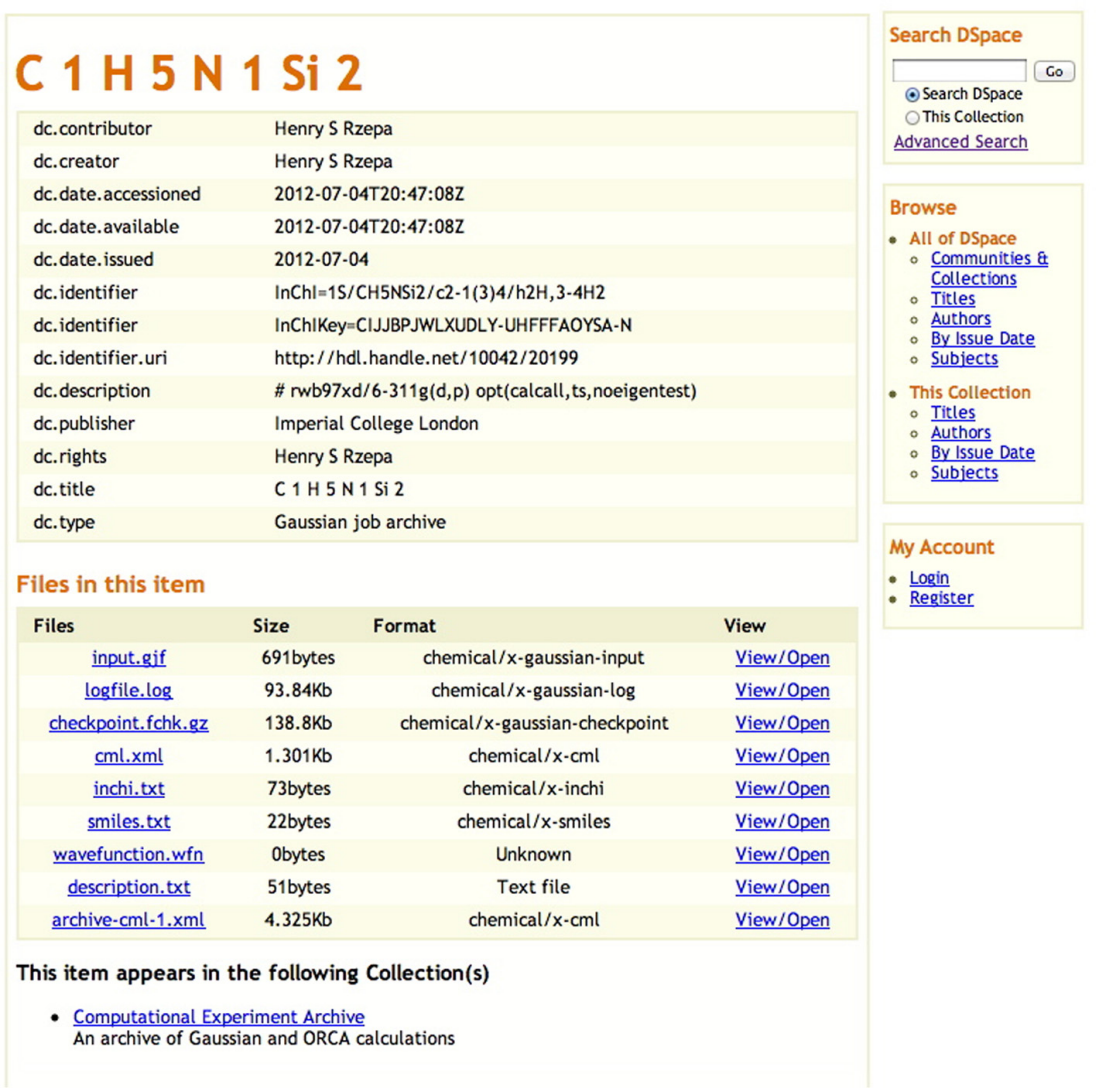
**A data repository entry in DSpace, showing associated chemical metadata.** The original can be retrieved at handle: 10042/20199.

**Figure 4:**
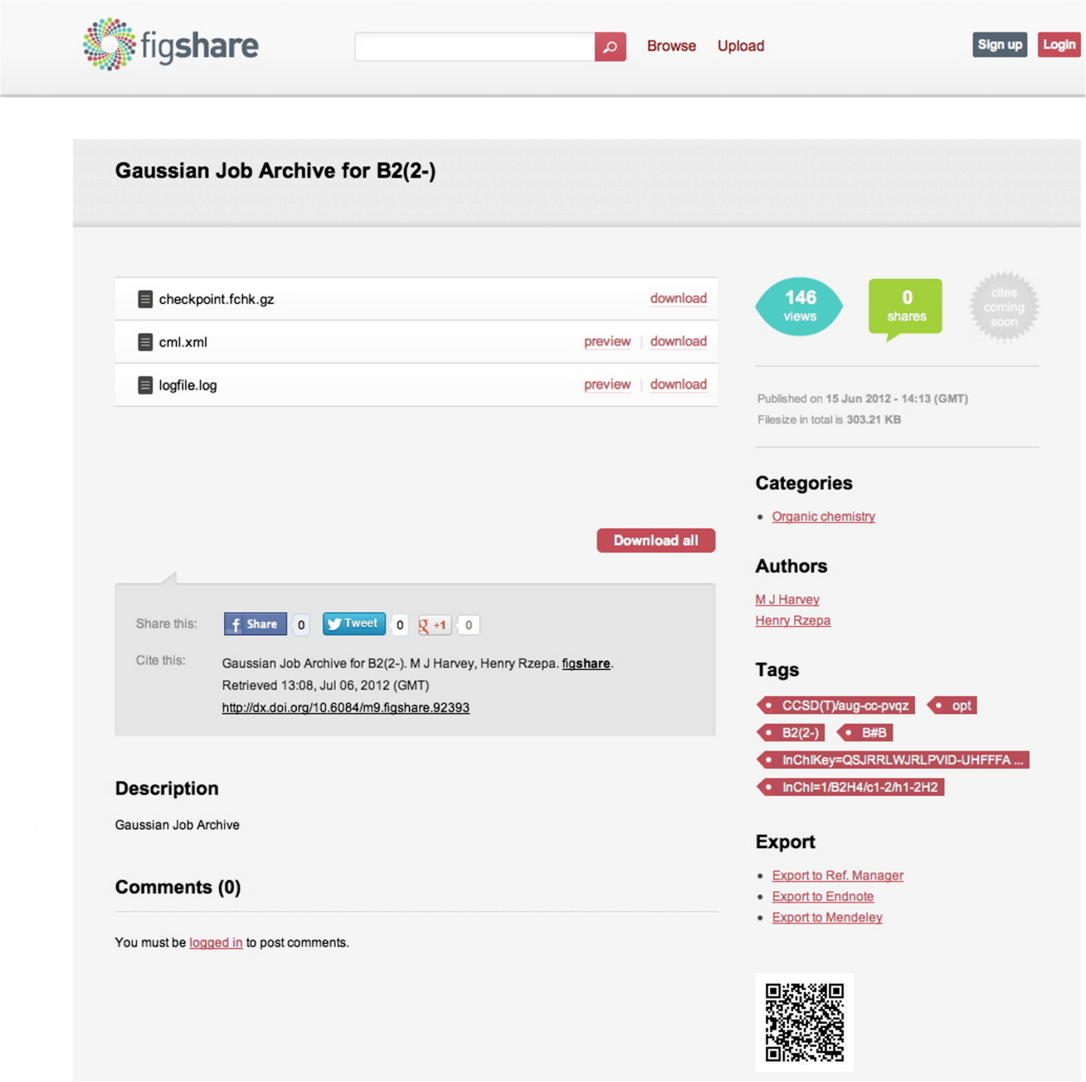
**A data repository entry in Figshare, showing associated chemical metadata.** The original can be retrieved at doi: http://dx.doi.org/10.6084/m9.figshare.95816.

1. The DSpace-based SPECTRa repository [24]. Each entry here is created from raw data files, and the metadata is added by post-processed recognition of regular patterns in the data, along with meta-data captured from the user or system at time of deposition. The resulting data-collection is identified with a unique handle, which can be resolved by the same resource as the digital object identifier (DOI) now ubiquitously used in journal articles such as the one you are current reading. A typical set of meta-data and raw data for the type of calculation reported in this article can be seen in Figure 3. The raw files themselves are associated with appropriate MIME types [25] to enable automated processing when downloaded. The entire collection is created automatically from the job submission portal used to create the data in the first place, ensuring it is as free of human error as possible. The unique molecule identifiers (the InChIKey) are captured as assigned to Dublin-core fields, and can also serve well as nodes in an RDF description [26], although Dspace itself cannot be used to invoke a semantic query based on such RDF declarations. 

2. Figshare [27] is a new, more general data repository, carrying much the same meta-data as the DSpace example (Figure 4). It too associates a DOI with the data set, and can be used in the same manner. 

3. ChemPound [28] was designed specifically to archive chemical information, and generates meta-data for RDF declaration at a much more finely grained level. For example, the final total energy in a quantum mechanical calculation is identified and associated with an RDF triple. The chempound repository is also the only one specifically designed for RDF-SPARQL like semantic queries of the triple store. Unlike the first two repositories however, Chempound does not (yet) generate a unique handle for identification of each entry.

#### Examples of digital repositories containing other types of data

Examples of other projects for depositing and curating data include DataOne [29], Dryad [30] (another DSpace-based repository), DataCite [31] (which also provides DOI identifiers for each collection) and DataShare (an online digital repository of multi-disciplinary research datasets produced at the University of Edinburgh) [32]. There are also separate initiatives for developing standards for the deposition and searching of data [33]. It is becoming clear that such repositories are bifurcating into two types; those for general data that carry only general meta-data descriptors for the content, and subject-specific repositories which serve to harvest much more finely tuned meta-data, in turn allowing much more specific searches of the repository to be made. If the fragmentation into increasingly subject-specific content continues, then the challenge will refocus on searching across different repositories for related data sets between which there may be valuable synergies.

Two examples of how such a strategy may be deployed are discussed next.

### The Java-based datument

Transclusion of (chemical) data-objects into HTML pages for humans to read has evolved in three phases. One of the earliest was introduced around 1996 and benefited from the close physical proximity in San Francisco of two commercial organisations, Netscape and MDL Ltd and the earlier publication of an article on the topic [34]. The reader had to download the software (Chime) and install it each computer they wished to use. This was replaced a few years later by the use of Java, whereby the necessary software archive (.jar) is downloaded automatically when the data-object is loaded. This mode is used to the present day on most conventional operating systems and is illustrated below. It makes use of a digitally signed .jar file, which allows data to be extracted from the display by the user (hence the prompt the user receives to accept the datument source when it is loaded). An example of this is illustrated in Additional file 1: Interactivity box 1. 

### The HTML5 based datument

This mode of presentation takes advantage of the new generation of mobile devices such as touch-screen tablets. It also presents a strategy for browser and device independence, since there does seem to be a trend towards increased adoption of standards centered around HTML5 for both browsers and the devices they run on. In this regard, the design of mobile devices appears to be evolving away from dependency on power-consuming software environments such as Java, and towards data-handling environments such as JSON [35] and Javascript, utilising lightweight graphics renderings based on WebGL in combination with HTML5 that can take full advantage of such an environment. An advantage over a Java-based solution is that the necessary display code is much smaller and runs natively within the browser rather than as a Java virtual environment. Two such implementations for HTML5 are ChemDoodle [36] and GLMol [37], for which examples of different types of transcluded data Additional files 2,3,4 (Interactivity Boxes) are shown below [38]. Data can also be flexibly retrieved from such objects [39]. A comparison between the static Figures 1 and 2 and the data-rich interactivity boxes serves to illustrate how an enhanced perception by reader can be achieved when they are allowed to interact with the datument. 

### The authoring perspective

I should also describe the experience of creating such figures from an author's perspective. The data-carrying components are embedded in the form of scripts, which themselves are can be regarded by the author as (publisher-provided?) templates, and the only real task is to provide appropriate variable names.

1. Additional file 1: Interactivity box 1 is created using a script for a device-sensitive display, which supports either a Java-based Jmol applet, or a Javascript-based ChemDoodle canvas: <script type="text/javascript" title="Script for creating a Canvas with device-sensitive display"> Figure 3 = Jmol.getApplet("Figure 3", Info1) Jmol.script (Figure 3,"load dna_mo148.cub.xyz;background image 'helix-back.jpg';spin 5;#alt:LOAD dna_mo148.cub.xyz") </script>

2. The links in the interactivity box of Figure 3 are created as: <a href="javascript:Jmol.loadFile(Figure 3,'1ZNA-H.mol',';background%20image%20"helix-back.jpg";measure%2083%20114;measure%20124%20155; measure%2045%2076;measure%204%2035;write%20jmol%20Figure3-1.jmol;')">(View/download model)</a>

3. Additional file 2: Interactivity box 2 is created using just a Javascript-based ChemDoodle canvas; <script type="text/javascript" id="a1"> if(ChemDoodle.featureDetection.supports_webgl()){ var transformBallAndStick1 = new ChemDoodle.TransformCanvas3D('transformBallAndStick1', 550, 450); transformBallAndStick1.specs.projectionWidthHeightRatio_3D = 550 / 450; transformBallAndStick1.specs.set3DRepresentation('Ball and Stick');

4. TransformBallAndStick1.specs.backgroundColor = 'white'; var molFile = httpGet('**datument.mol**'); var molecule = ChemDoodle.readMOL(molFile,2); transformBallAndStick1.loadMolecule(molecule); }else{document.write('<img src="Figure 4.jpg" />');} </script>

The important variables in the above are simply the names of the data file (*e.g. datument.mol*). Other important attributes such as the size of the canvas etc. can be defined using information arrays (*e.g.* Info1). Such scripts are easily wrapped into *e.g.* HTML5 components such as *widgets* (interactive, and potentially 3D objects), which in turn can be absorbed into authoring environments (such as iBooks author) [40] as transcluded objects, a category that also includes tables, charts and image media. Whilst the average compositor of a scientific article is currently well acquainted with the latter type, familiarity with the concept of including *e.g.* a data-handling widget may well become a skill essential to authoring the science article of the future. Perhaps the most realistic starting point might be to encourage (require?) Ph.D. theses to be prepared and examined in such enhanced formats. Certainly it is increasingly a requirement imposed by examiners to have available the data underlying the theses in digita, easily viewed form. It is also becoming accepted that theses can contain DOI resolvers to pertinent data-sets supporting the research being examined. The conversion of such material into journal articles might not then appear a challenge.

## Conclusions

The ever increasing molecular complexity of modern chemistry presents interesting new challenges for how the underlying models may best be shared between scientists. A researcher should not have to use what may amount to inspired guesswork to reconstitute such a model from a journal article. Here I have taken two examples of complex molecular structures and by embedding descriptive data within this article, have created a working tool, a datument for the researcher. I noted earlier that one of the issues that needs addressing is whether the necessary tools for doing so would be accessible for the average scientific author. This particular datument was in fact written and assembled over a period of two days, although several of its components were already available (having been prepared as part of teaching notes on conformational analysis for lectures delivered by the author). Higher order tools (such as Apple iBooks author [40]) show how some of the functionality needed could be absorbed into a simple to use tool. Another source of publishable datuments might come from the new generations of electronic laboratory notebooks in chemistry, and these are also increasingly interfacing to digital repositories.

There are also signs that after a long induction period, some publishers are starting to adopt such technologies for journal publication. But there are also dangers. For example, will a datument simply come to be treated as a rights-managed document, with both the full text and the data ardently protected by the publisher's commercial model? Will such enriched publications result in significantly more expensive journals? Will publishers allow datuments to be mined for their data by software agents [20] such as OSCAR? And can a datument be appropriately curated to ensure accessibility long into the future? These are important issues, but we must ensure that resolution includes active participation from both the authors of scientific datuments and their consumers.

## Competing interests

The author declares that they have no competing interests.

## Supplementary Material

Additional file 1 **Interactivity box 1.**^a^ Data-based object illustrating various aspects of the interaction at the heart of Z-DNA. Publisher note: Due to the Publisher’s current document type definition it is necessary that the author’s Interactivity box files are labeled "Additional file". Click here for file

Additional file 2 **Interactivity box 2.**^a^ Data-rich molecular model rendered using ChemDoodle, illustrating one structure involved in the co-polymerisation of carbon dioxide and cyclohexene epoxide. Publisher note: Due to the Publisher’s current document type definition it is necessary that the author’s Interactivity box files are labeled "Additional file". Please also note: Without WebGL enabled, this will appear as a static image only. Click here for file

Additional file 3 **Interactivity box 3.**^a^ Data-rich IR spectrum rendered 526 in the datument using ChemDoodle. Publisher note: Due to the Publisher’s current document type definition it is necessary that the author’s Interactivity box files are labeled "Additional file". Click here for file

Additional file 4 **Interactivity box 4.**^a^ Data-rich molecular coordinate 528 model created using GLMol,^36^ illustrating one structure involved in the 529 co-polymerisation of carbon dioxide and cyclohexene epoxide. Publisher note: Due to the Publisher’s current document type definition it is necessary that the author’s Interactivity box files are labeled "Additional file". Please also note: Without WebGL enabled, this will appear as a static image only. Click here for file
