# Supplementary material for: Chemical datuments as scientific enablers
Source: J Cheminform. 2013 Jan 23;5:6. doi: 10.1186/1758-2946-5-6 (PMC3552767; doi:10.1186/1758-2946-5-6)
Supplement: Additional file 1 — Interactivity box 1.a Data-based object illustrating various aspects of the interaction at the heart of Z-DNA. Publisher note: Due to the Publisher’s current document type definition it is necessary that the author’s Interactivity box files are labeled "Additional file". [file 1758-2946-5-6-S1.zip › index.html]

Chemical datuments as scientific enablers.


| *Interactivity box 1:*a Data-based object illustrating various aspects of the interactions at the heart of Z-DNA. | |
| --- | --- |
|  |  |
| --- | --- |
| a The model includes two bases, two furanose rings linked by a phosphate, of which the charge is balanced by an ammonium cation. Data must be loaded by invoking the hyperlinks present in the preceding text. To invoke the **user menu**, right (or ctrl) click in the viewing area, a feature which can also be used to save the model data. The original complete data set is also available at http://dx.doi.org/10042/to-13541 *via* a digital repository. bThe interactive components are created by invoking the Java-based Jmol applet in the browser window. If your browser does not support Java, the display will instead default to using ChemDoodle. This currently does not support the scripted interactive functionality and only the model coordinates will be loaded. c This file is downloaded as a ZIP-compressed archive containing the components required to reconstitute the scene. | |
| We will pose the question: what are the origins of the relative stability of the left handed CG-rich duplex (View/download model)b,c compared with the isomeric right handed helix? The following are some of the chemically interesting interactions that can be identified at the heart of the system:   1. A furanose oxygen approaches to within 2.85Å of a guanine ring 2. A furanose oxygen approaches to within  alt load of 2.85Å of a guanine ring 3. A furanose OC-H hydrogen is identified within 2.48Å of a second furanose oxygen 4. This hydrogen bond is facilitated by a C-Hσ (BD)/C-Oσ\* (BD\*) anti-periplanar alignment, which results in acidification of the C-H bond (shown as magenta bonds). The NBO interaction energy is **E(2) 5.8** kcal mol-1 which may be considered of a chemically significant magnitude. 5. The is an anomeric interaction between guanine and the ribose. The overlap and interaction between the NLp and C-Oσ\* orbitals results in a NBO perturbation energy of E(2) of 11.6 kcal mol-1. The colour coding of the NBO orbital isosurface (contoured at 0.02au) indicates a positive orbital overlap between the blue and purple surfaces (of the acceptor and donor orbitals respectively), and likewise between the red and orange surfaces. This stereoelectronic interaction helps to rigidify the orientation of the plane of the nitrogen base with respect to the sugar ring, and might be expected to influence the higher order structures of the polymer such as supercoiling and unwinding. 6. There is a further rather weaker anomeric interaction in a cytosine-furanose pair (**E2 6.8**). 7. A *Gauche*-like conformation of the ethane-1,2-diol fragment (gold bond) is also aided by a stereoelectronic interaction between the OLp and an adjacent C-Oσ\* bond. 8. A non-covalent-interaction (NCI) analysis reveals further weaker interactions in this system, which might otherwise be overlooked. The isosurface colour coding maps to blue = attractive, green = weakly attractive, yellow = weakly repulsive and red = strongly repulsive interactions 9. Data can be retrieved from this object by invoking the user menu or an appropriate script.   Mindful that all these interactions are repeated throughout the DNA polymer, their accumulation might be expected to influence the higher order structures of this important biopolymer. |  |
