# Supplementary material for: Chemical datuments as scientific enablers
Source: J Cheminform. 2013 Jan 23;5:6. doi: 10.1186/1758-2946-5-6 (PMC3552767; doi:10.1186/1758-2946-5-6)
Supplement: Additional file 1 — Interactivity box 1.a Data-based object illustrating various aspects of the interaction at the heart of Z-DNA. Publisher note: Due to the Publisher’s current document type definition it is necessary that the author’s Interactivity box files are labeled "Additional file". [file 1758-2946-5-6-S1.zip › test.html]

Jmol/ChemDoodle Demo -- Using JmolData.jar for serving ChemDoodle models
