# Supplementary material for: Chemical datuments as scientific enablers
Source: J Cheminform. 2013 Jan 23;5:6. doi: 10.1186/1758-2946-5-6 (PMC3552767; doi:10.1186/1758-2946-5-6)
Supplement: Additional file 2 — Interactivity box 2.a Data-rich molecular model rendered using ChemDoodle, illustrating one structure involved in the co-polymerisation of carbon dioxide and cyclohexene epoxide. Publisher note: Due to the Publisher’s current document type definition it is necessary that the author’s Interactivity box files are labeled "Additional file". Please also note: Without WebGL enabled, this will appear as a static image only. [file 1758-2946-5-6-S2.zip › index.html]

Chemical datuments as scientific enablers. Interactivity box 2.


| *Interactivity box 2.*a Data-rich molecular model rendered using ChemDoodle, illustrating one structure involved in the co-polymerisation of carbon dioxide and cyclohexene epoxide. |
| --- |
|  |
| --- |
| aThe full data for the object above can be inspected at http://dx.doi.org/10042/to-8229 |
|  |
