# Supplementary material for: Chemical datuments as scientific enablers
Source: J Cheminform. 2013 Jan 23;5:6. doi: 10.1186/1758-2946-5-6 (PMC3552767; doi:10.1186/1758-2946-5-6)
Supplement: Additional file 3 — Interactivity box 3.a Data-rich IR spectrum rendered 526 in the datument using ChemDoodle. Publisher note: Due to the Publisher’s current document type definition it is necessary that the author’s Interactivity box files are labeled "Additional file". [file 1758-2946-5-6-S3.zip › index.html]

Chemical datuments as scientific enablers. Box 3.


| *Interactivity box 3.*a Data-rich IR spectrum rendered in the datument using ChemDoodle. |
| --- |
|  |
| --- |
| aThe spectrum can be expanded by suitable "gestures" |
|  |
